# Rapid intraoperative molecular genetic classification of gliomas using Raman spectroscopy

**DOI:** 10.1093/noajnl/vdz008

**Published:** 2019-05-28

**Authors:** Laurent James Livermore, Martin Isabelle, Ian Mac Bell, Connor Scott, John Walsby-Tickle, Joan Gannon, Puneet Plaha, Claire Vallance, Olaf Ansorge

**Affiliations:** 1 Nuffield Department of Clinical Neurosciences, John Radcliffe Hospital, University of Oxford, UK; 2 Renishaw plc., Spectroscopy Products Division, UK; 3 Department of Chemistry, University of Oxford, UK

**Keywords:** brain tumor, intraoperative, isocitrate hydrogenase, Raman spectroscopy, personalized medicine

## Abstract

**Background:**

The molecular genetic classification of gliomas, particularly the identification of isocitrate dehydrogenase (IDH) mutations, is critical for clinical and surgical decision-making. Raman spectroscopy probes the unique molecular vibrations of a sample to accurately characterize its molecular composition. No sample processing is required allowing for rapid analysis of tissue. The aim of this study was to evaluate the ability of Raman spectroscopy to rapidly identify the common molecular genetic subtypes of diffuse glioma in the neurosurgical setting using fresh biopsy tissue. In addition, classification models were built using cryosections, formalin-fixed paraffin-embedded (FFPE) sections and LN-18 (IDH-mutated and wild-type parental cell) glioma cell lines.

**Methods:**

Fresh tissue, straight from neurosurgical theatres, underwent Raman analysis and classification into astrocytoma, IDH-wild-type; astrocytoma, IDH-mutant; or oligodendroglioma. The genetic subtype was confirmed on a parallel section using immunohistochemistry and targeted genetic sequencing.

**Results:**

Fresh tissue samples from 62 patients were collected (36 astrocytoma, IDH-wild-type; 21 astrocytoma, IDH-mutated; 5 oligodendroglioma). A principal component analysis fed linear discriminant analysis classification model demonstrated 79%–94% sensitivity and 90%–100% specificity for predicting the 3 glioma genetic subtypes. For the prediction of IDH mutation alone, the model gave 91% sensitivity and 95% specificity. Seventy-nine cryosections, 120 FFPE samples, and LN18 cells were also successfully classified. Meantime for Raman data collection was 9.5 min in the fresh tissue samples, with the process from intraoperative biopsy to genetic classification taking under 15 min.

**Conclusion:**

These data demonstrate that Raman spectroscopy can be used for the rapid, intraoperative, classification of gliomas into common genetic subtypes.

**Key points**

1. Raman spectroscopy can be used for rapid, intraoperative, classification of gliomas.
2. Simple intraoperative protocol with results in under 15 min.
3. Accurate genetic classification possible with cryosections and FFPE sections.

**Importance of the Study**

In this era of personalized medicine and the development of treatments targeting specific glioma genetic subtypes, it will become increasingly important for surgeons to be aware of the genetics of a tumor at the time of operation to inform their surgical strategy and to balance the survival benefit of increasing surgical resection with the risk of causing neurological deficit. In this study, we demonstrate that Raman spectroscopy can accurately and rapidly classify gliomas according to isocitrate dehydrogenase mutations and 1p/19q-codeletion. The protocol can be carried out within a neurosurgical theatre without the need for laboratory input and results are available in under 15 min.

Diffuse gliomas are the most common and aggressive primary tumors of the central nervous system in adults. The molecular genetic stratification of gliomas is important as more evidence emerges of the predictive and prognostic implications of different genetic subtypes. Surgical resection plays a key role in the management of patients with gliomas, and there is increasing evidence that the greater the extent of surgical resection, the greater the patient’s progression-free and overall survival.^1–3^ The diffuse nature of these tumors means that the surgeon can never achieve complete tumor resection. At present, it has been demonstrated that the clinical benefit of the extent of tumor resection does depend on the specific genetic subtypes.^2^ Information on the tumor genetic subtype at the time of surgery, providing real-time predictive and prognostic information, would allow the surgeon to tailor their surgical strategy to each patient’s genetic profile.

The 2016 World Health Organization (WHO) classification of gliomas introduced molecular and genetic subtyping integrated with the histological glioma type and grade^4^ (Figure 1). Common and clinically relevant genetic subtypes include gliomas with mutations of the isocitrate dehydrogenase (IDH) genes 1 or 2 with or without chromosome 1p and 19q (1p/19q) codeletion.^5^ Within most neuropathology laboratories, 1p/19q-codeletion is established using the cytogenetic technique of fluorescence in-situ hybridization (FISH). The presence of the most common IDH mutation (IDH1 R132H) can be established using immunohistochemistry (IHC) with an antibody highly sensitive and specific for this IDH mutation,^6^ which accounts for around 85%–90% of the mutations in gliomas.^7^ If there is suspicion of a rare IDH mutation, the sample would undergo targeted IDH-1/IDH-2 genetic sequencing.

**Figure 1.**
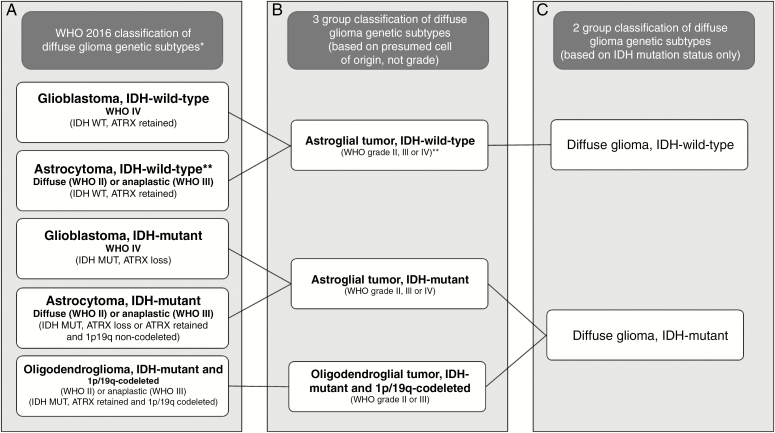
(A) The World Health Organization (WHO) 2016 classification of diffuse gliomas; (B) genetic subtypes for 3-group model; (C) genetic subtypes for 2-group model. (*not all subtypes are shown, only common groups; ** these are provisional categories in the classification: it is now recognized that most of these neoplasms are incipient or undersampled glioblastomas, isocitrate dehydrogenase-wild-type^11^).

Raman spectroscopy is a vibrational spectroscopy technique that probes the unique molecular vibrations of a sample to characterize its molecular composition.^8^ The significant advantage of Raman analysis is that no or minimal sample processing is required, allowing for rapid and cost-effective analysis of tissue without the need for specialist staff, time-consuming laboratory protocols, or expensive consumables.

The main aim of this study was to evaluate the ability of Raman spectroscopy to identify the most common molecular genetic subtypes of diffuse glioma in the neurosurgical theatre setting. An important objective was to develop a simple protocol, which would allow the surgeon or scrub staff to undertake Raman analysis of tissue in the operating theatre without the need for specialized technicians or laboratories. We have also evaluated the application of Raman analysis in the neuropathological diagnostic workflow of glioma tissue, including analysis of cryosections, formalin-fixed paraffin-embedded (FFPE) sections, and glioma cell lines.

## Materials and Methods

### Study Participants

Ethical approval for the use of fresh, cryosection, and FFPE tissue was obtained through the Oxford Brain Bank generic Research Ethics Committee approval (15/SC/0639). All tissue used was deemed surplus to diagnostic requirements.

### Pathological Diagnosis

All cases were reviewed by a consultant neuropathologist, and diagnoses were established using the European Association for Neuro-Oncology guidelines,^9^ which incorporates the updated (2016) WHO classification of tumors of the central nervous system.^4^ See Supplementary material for details of histopathological diagnostic protocols. The WHO integrated diagnosis was based on the IHC/genetic results and histological grade (Figure 1), giving following 5 possible diagnostic groups:

1. diffuse or anaplastic astrocytoma, IDH-mutant (grade II or III);
2. glioblastoma, IDH-mutant (grade IV);
3. oligodendroglioma or anaplastic oligodendroglioma, IDH-mutant, and 1p/19q-codeleted (grade II or III);
4. diffuse or anaplastic astrocytoma, IDH-wild-type (grade II or III);
5. glioblastoma, IDH-wild-type (grade IV).

### Tissue Processing

**Figure 2.**
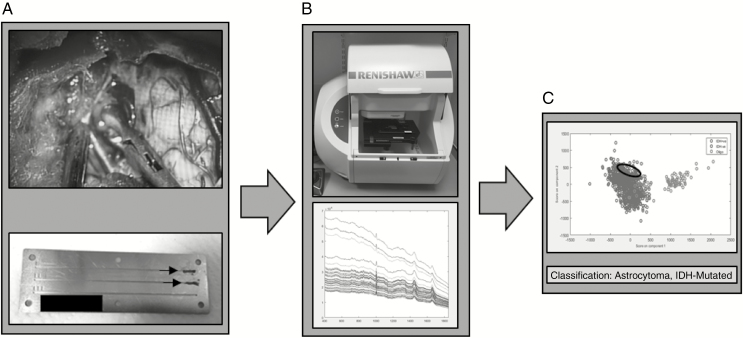
Fresh tissue workflow. (A) Intraoperative fresh tissue biopsy squashed into grooved stainless steel slide (arrows). (B) Slide placed in Raman spectrometer and multiple spectra acquired. (C) Spectra classified into genetic subtype using principal component analysis fed linear discriminant analysis model.

For fresh tissue samples, 1 biopsy per patient was taken by the neurosurgeon from an area of tumor bulk defined both visually and using the intraoperative neuronavigation system (Brainlab, Germany). The biopsy was immediately transferred to the neuropathologist, who cut the tissue biopsy into 2 pieces: 1 for intraoperative smear evaluation, and the adjacent piece for Raman analysis. The portion for Raman analysis measured approximately 3 mm^3^. The sample was immediately transferred to a grooved stainless steel slide^10^ (Figure 2) and compressed into the groove using a surgical scalpel creating a relatively flat surface onto which the Raman laser could be focused and compacting the tissue to limit the effect of tissue drying. The adjacent biopsy underwent intraoperative smear preparation with hematoxylin and eosin (H&E) and was reviewed by the consultant neuropathologist to confirm the presence of tumor.

Details of cryosection, FFPE, and LN18 cell line preparation can be found in the Supplementary material. Two LN18 IDH-mutant and 2 IDH-wild-type cell lines were cultured and processed separately at 2 different time points. Cell line batches, once harvested, were processed as either cryosections or FFPE sections, with protocols to match the respective tissue sample preparations.

### Raman Spectroscopy

Raman spectra were collected using a Renishaw bench-top RA800 series spectrometer (Renishaw plc., UK). For fresh tissue samples, single point spectra were obtained, widely distributed across the smeared sample, aiming for at least 100 spectra per fresh tissue sample. Two accumulations of either 0.5 s or 1 s depending on the amount of background fluorescence and resulting detector saturation for that sample. The total time to acquire data from each area was taken from the spectrometer log. The time from surgical biopsy to completion of spectral collection was also recorded. See Supplementary material for further details.

### Glioma Genetic Subtype Models

For each tissue preparation type, 2 separate models were built. The first was a 2-group model, stratified based on the IDH mutation alone (Figure 1C):

1. IDH-mutant (including all IDH-mutant astrocytomas, glioblastomas, and oligodendrogliomas);
2. IDH-wild-type (including all IDH-wild-type diffuse astrocytomas and glioblastomas).

The second model was a 3-group model, stratifying gliomas into the 3 main genetic subtypes (Figure 1B) regardless of histological grade:

1. astrocytomas, IDH-mutant (astrocytoma [grade II or III] and glioblastoma [grade IV]);
2. astrocytomas, IDH-wild-type (astrocytoma [grade II or III] and glioblastoma [grade IV]);
3. oligodendrogliomas, IDH-mutant, and 1p/19q-codeleted (grade II or III).

All astrocytomas, IDH-wild-type were glioblastomas (grade IV)—there were no grade II or III astrocytomas, IDH-wild-type tumors; these are often undersampled glioblastomas.^11^

### Statistical Analysis and Model Building

All data analysis was carried out using MATLAB R2015b (MathsWorks). For each genetic subtype, normalized (using Extended Multiplicative Scatter Correction^12^) mean spectra and the negative of the second derivative^13^ were plotted against the wave number shift. Statistical differences between peak intensities were evaluated using the Wilcoxon rank-sum test, and the magnitude of the resulting *P* value was used as an indicator of the magnitude of the difference between the intensities. Tentative molecular assignment of the identified Raman peaks was carried out based on the published literature.^14–16^ See Supplementary material for further details of spectral preprocessing and analysis.

Principal component analysis (PCA) fed linear discriminant analysis (LDA) was used to build the pathology classification models, which used all the spectra, after preprocessing, from each patient. In this classification method, PCA is used to reduce the data dimensionality, with the analysis of variance used to select only those PCs that describe a statistically significant difference in the data. An LDA model is then built from those significant PCs. Model performance was assessed using leave-one-patient-out cross-validation to generate model sensitivity and specificity (Supplementary material). Multi-class receiver operating characteristic (ROC) curves were generated with the area under ROC curve (AUROC) calculated as a measure of accuracy.

## Results

A total of 62 fresh tissue samples were included in the study. No cases were excluded on the grounds of the absence of tumor on the adjacent H&E analysis of the smeared section. Ninety-seven cryosections and 132 FFPE sections were cut for analysis. Of these, 18 cryosections and 12 FFPE sections were excluded after review of the parallel H&E section showed no evidence of tumor. A total of 79 cryosections and 120 FFPE sections were used for analysis. Table 1 shows the number of samples analyzed in each genetic subgroup for each sample preparation method.

**Table 1.** Number of Each Genetic Subtype Included for Fresh Tissue, Cryosections and Formalin-Fixed Paraffin-Embedded (FFPE) Sections, Subdivided into 3-Group Model and 2-Group Model

Genetic subtypes (WHO grade)	Tissue preparation					
	Fresh tissue		Cryosections		FFPE sections	
	3-grp model	2-grp model	3-grp model	2-grp model	3-grp model	2-grp model
Astrocytoma, IDH-wild-type (All WHO IV (GB))	36	36	19	19	41	41
Astrocytoma, IDH-mutant (WHO II/WHO III/WHO IV (GB))	21 (4/7/10)	26	41 (12/10/19)	60	51 (8/12/31)	79
Oligodendroglioma (WHO II/WHO III)	5 (3/2)		19 (8/11)		28 (12/16)	
Total	62		79		120	

GB = glioblastoma; grp = group; IDH = isocitrate dehydrogenase; WHO = World Health Organization.

A total of 9799 spectra were collected in the fresh tissue group, with a mean of 161 spectra per sample. The mean time for data collection was 6 min and 6 s per sample, and the whole process from intraoperative biopsy to completion of Raman spectroscopy was under 15 min in every case. See Supplementary material for cryosection and FFPE details).

### Classification Model Performance

**Figure 3.**
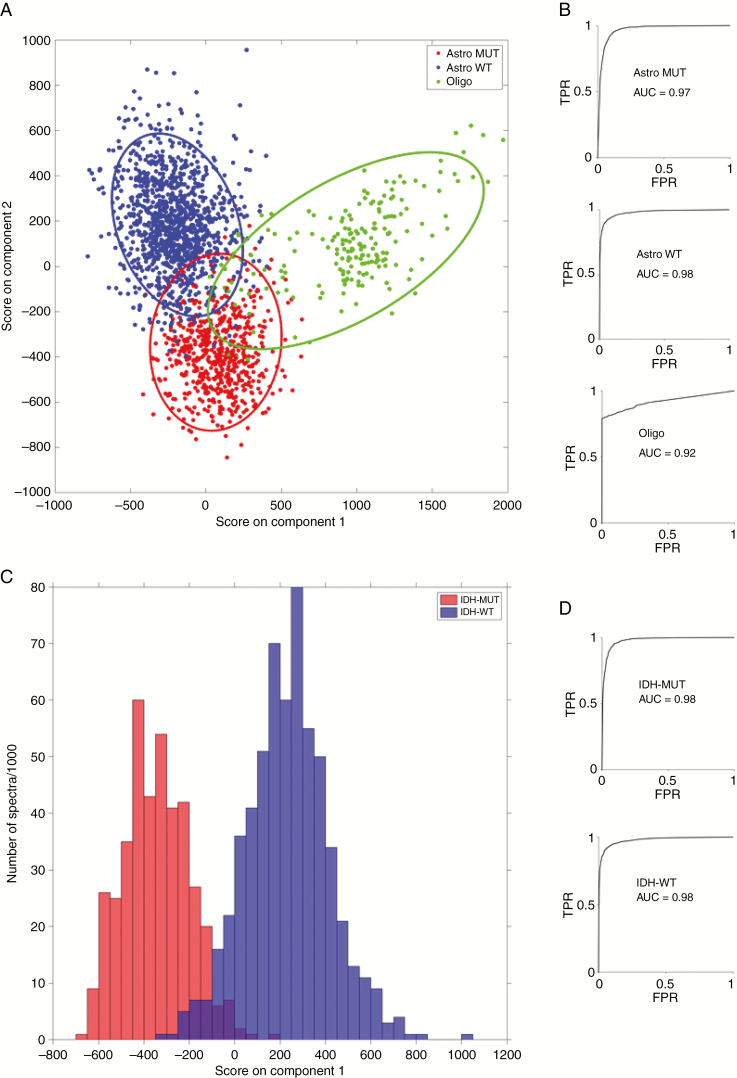
Fresh tissue linear discriminant analysis score plots and receiver operating characteristic curves for 3-group model (A and B) and 2-group model (C and D). (TPR = true positive rate; FPR = false positive rate; Astro MUT = astroglial tumor isocitrate dehydrogenase [IDH]-mutant; Astro WT = astroglial tumor, IDH-wild-type; Oligo = Oligodendroglioma).

Table 2 summarizes the sensitivities and specificities for 3-group and 2-group prediction of glioma genetic subtypes using PCA-LDA classification modeling of the Raman spectra with leave-one-patient-out cross-validation. With fresh tissue samples, the models were able to classify tumors into either IDH-mutant or IDH-wild-type (2-group model) with 0.91–0.95 sensitivity and specificity and 0.98 accuracy. Fresh tissue classification into the 3 genetic subtypes (3-group model) was performed with 0.79–0.94 sensitivity, 0.89–1.00 specificity, and an accuracy of between 0.92 and 0.98 (Figure 3). Cryosection and FFPE section models classified with slightly less sensitivity and specificity than the fresh tissue models as shown in Table 2. The overall accuracy of these models remained high, with cryosection 3-group classification accuracy of 0.89–0.93, cryosection 2-group classification accuracy of 0.93 (Supplementary Figure 4), FFPE section 3-group classification accuracy of 0.87–0.93, and FFPE section 2-group classification accuracy of 0.82 (Supplementary Figure 5). LDA plots for the 3-group and 2-group models show good separation of the genetic subtypes using fresh tissue (Figure 3), cryosections (Supplementary Figure 4), and FFPE sections (Supplementary Figure 5).

**Table 2.** Sensitivity and Specificity for Predicting Genetic Subtypes Using PC-Linear Discriminant Analysis (LDA) 3-Group and 2-Group Models for Fresh Tissue Samples, Cryosections, and Formalin-Fixed Paraffin-Embedded (FFPE) Sections

Model	Genetic subtypes	Fresh tissue		Cryosections		FFPE sections	
		Sensitivity	Specificity	Sensitivity	Specificity	Sensitivity	Specificity
3-group	Astrocytoma, IDH-wild-type	0.94	0.90	0.78	0.85	0.81	0.84
	Astrocytoma, IDH-mutant	0.91	0.95	0.79	0.89	0.72	0.87
	Oligodendroglioma	0.79	1.00	0.74	0.90	0.79	0.93
2-group	IDH-wild-type	0.95	0.91	0.88	0.83	0.77	0.71
	IDH-mutant	0.91	0.95	0.83	0.88	0.71	0.77

### Mean Spectra and Peak Assignment

**Figure 4.**
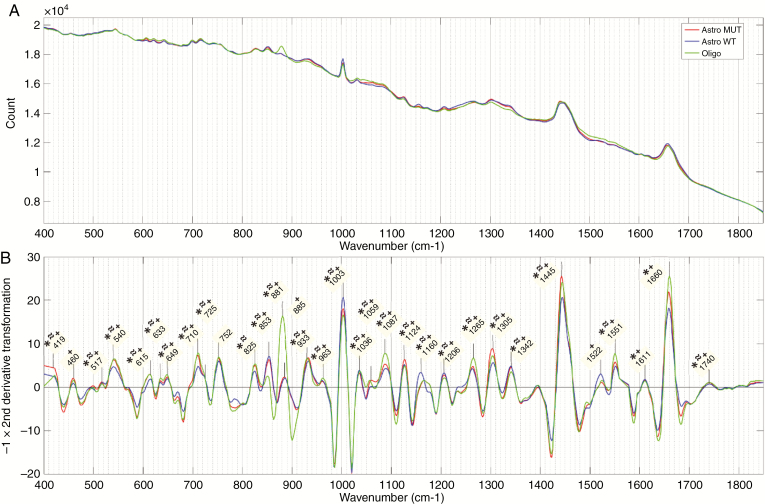
Fresh tissue 3-group model. (A) Mean spectra and (B) negative of second derivative transformation for each genetic subtype. Prominent Raman peaks labeled in panel B. Statistical significance between peak intensities of each genetic subtype in the model is indicated (*P* < .01) for *isocitrate dehydrogenase (IDH)-mutant versus IDH-wild-type; ^≈^Astro MUT versus Oligo; and ⁺Astro wild-type (WT) versus Oligo. (Astro MUT = astroglial tumor, IDH-mutant; Astro WT = astroglial tumor, IDH-wild-type; Oligo = Oligodendroglioma).

**Figure 5.**
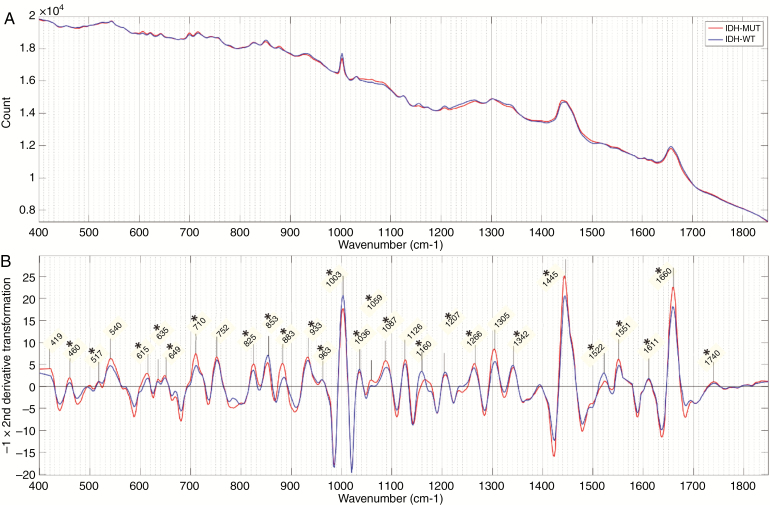
Fresh tissue 2-group model. (A) Mean spectra and (B) negative of the second derivative transformation of mean spectra for each genetic subtype. Prominent Raman peaks labeled in panel B. Statistical significance between peak intensities of each genetic subtype is indicated (*P* < .01) for *isocitrate dehydrogenase (IDH)-mutant versus IDH-wild-type. (IDH-MUT = IDH-mutant; IDH-WT = IDH-wild-type).

For the fresh tissue samples, mean spectra and the negative of the second derivative of the mean spectra for each genetic subgroup in the 3-group model are shown in Figure 4. Similar figures for cryosection and FFPE sections are shown in Supplementary Figure 1. For the 2-group model, mean spectra and the negative of the second derivative of the mean spectra are shown in Figure 5 for the fresh tissue samples and Supplementary Figure 2 for cryosections and FFPE sections. On all second-derivative plots, the wave numbers of prominent Raman peaks are labeled and the statistical significance of the difference between the peak intensities of each genetic subtype in that model is indicated (*P* < .001, Wilcoxon rank-sum test).

The most dominant Raman peaks in the fresh tissue sample are seen at 1445 cm^–1^ and 1660 cm^–1^. These are both a summation of multiple overlapping peaks and represent the ratio of protein and lipid content within the sample. There is also a characteristically narrow peak at 1003 cm^–1^, which, along with peaks at 615 cm^–1^ and 1036 cm^–1^_,_ represent phenylalanine. Other amino acid peaks include 460 cm^–1^ (tryptophan/tyrosine), 635 cm^–1^ and 649 cm^–1^ (tyrosine), and 540 cm^–1^ (cysteine). Various peaks were assigned to classes of lipids including triglycerides/fatty acids (853 cm^–1^, 1059 cm^–1^, 1087 cm^–1^, 1126 cm^–1^, 1160 cm^–1^, 1266 cm^–1^, 1305 cm^–1^, 1445 cm^–1^, 1740 cm^–1^) and cholesterol (419 cm^–1^, 825 cm^–1^). Peaks assigned to proteins include 1522 cm^–1^ and 1551 cm^–1^ (amide III) and 1660 cm^–1^ (amide I). DNA peaks were assigned at 517 cm^–1^, 883 cm^–1^, 963 cm^–1^, 1207 cm^–1^, and 1342 cm^–1^. Peaks at 710 cm^–1^ and 933 cm^–1^ most likely represent glycogen.

The majority of identified peaks exhibited significant differences in intensity between the various genetic subtypes. When comparing IDH-mutant with IDH-wild-type intensities, in both the 2- and 3-group fresh tissue models, the most significant differences (lowest *P* values) were found in peaks representing lipid (853 cm^–1^, 1059 cm^–1^, 1087 cm^–1^, 1266 cm^–1^, 1445 cm^–1^, and 1660 cm^–1^), phenylalanine (1003 cm^–1^), and DNA (1207 cm^–1^ and 1342 cm^–1^). The differences observed between astrocytomas, IDH-mutant, and oligodendroglioma were very similar to those observed between astrocytomas, IDH-wild-type, and oligodendroglioma with oligodendroglioma intensities having the most significant differences at peaks assigned to phenylalanine (1003 cm^–1^ and 1036 cm^–1^), DNA (881 cm^–1^, 885 cm^–1^, and 1342 cm^-–1^), and protein (1522 cm^–1^ and 1551 cm^–1^). The DNA peaks around 800–900 cm^–1^ are the most prominent of these. In general, the peak differences between oligodendroglioma and IDH-wild-type astrocytomas were more pronounced than between oligodendroglioma and IDH-mutant astrocytomas.

There is consistency between the dominant Raman peaks across the different tissue preparations. Depending on the model, between 12 and 17 peaks of the 43 peaks identified are present in all 3 tissue preparations. Of these, 11 peaks are also present in both LN18 preparations (cryosections and FFPE sections). In general, cryosection and FFPE section spectra have more prominent DNA and amino acid peaks and less contribution from lipid and protein peaks. The cryosection spectra have a significant background in the 1200–1500 cm^–1^ region, which is thought to be due to water condensation on the objective lens as the cryosection dries out. There is also a strong contribution from a prominent peak at 1336 cm^–1^ (assigned to protein:lipid ratios) in this wave number region, which is not seen in the fresh tissue spectra. In terms of differentiating between genetic subtypes, peaks that consistently give the most significant differences between subtypes across the 3 tissue preparations are those assigned to tyrosine (635 and 649 cm^–1^), phenylalanine (1003 cm^–1^ and 1036 cm^–1^), lipid (825 cm^–1^ and 853 cm^–1^), protein (1551 cm^–1^), and lipid:protein ratios (1445 cm^–1^:1551 cm^–1^). In the 3-group models including oligodendroglioma, peaks for DNA (725 cm^–1^, 963 cm^–1^, and 1206 cm^–1^) are also consistent across all sample preparations.

### Rare IDH

A total of 13 rare IDH mutations were present overall, in 3 fresh tissue samples, 6 cryosections, and 4 FFPE sections (Table 3). All cases were correctly classified into the IDH-mutated group (using the 2-group model), with the highest probability of correct classification occurring in the fresh tissue samples, with a mean probability of 91% for predicting IDH mutant.

**Table 3.** Rare Isocitrate Dehydrogenase (IDH) Mutations and Probability of Predicting IDH Mutation with Raman Spectroscopy (GB = glioblastoma)

Tissue preparation	Histology/grade	Rare IDH mutation confirmed with targeted genetic sequencing	Probability of predicting IDH mutation with Raman model
Fresh tissue	Astrocytoma (GB) IV	IDH1 394T	0.95
	Astrocytoma (GB) IV	IDH2 G515T	0.92
	Astrocytoma II	IDH2 G515A	0.87
Cryosections	Astrocytoma II	IDH1 R132C	0.92
	Astrocytoma II	IDH1 R132C	0.96
	Astrocytoma (GB) IV	IDH1 R394T	0.61
	Oligodengroglioma II	IDH2 R172K	0.93
	Oligodengroglioma II	IDH2 R172K	0.78
	Astrocytoma III	IDH1 R132C	0.77
FFPE sections	Astrocytoma (GB) IV	IDH1 R132C	0.57
	Astrocytoma (GB) IV	IDH1 R132G	0.82
	Oligodengroglioma II	IDH2 R172K	0.82
	Oligodengroglioma III	IDH2 R172W	0.89

### LN18 Cells

All LN18 IDH-mutant cell lines stained strongly for R132H IDH mutation on IHC. Mean spectra and the negative of the second derivative are shown in Supplementary Figure 3. The mean spectra for the LN18 cells and key Raman peaks, which differentiate genetic subtypes correlate with the tissue preparation spectra. Table 4 gives the sensitivity, specificity, and accuracy (AUROC) using PCA-LDA for each mutant and wild-type cell lines processed as cryosections or FFPE sections.

**Table 4.** LN18 Cell Lines. Sensitivity and Specificity for Predicting Isocitrate Dehydrogenase (IDH) Mutation Using PC-Linear Discriminant Analysis for Different Sample Preparation Methods Using Spectra From Individual Cell Line Batches and Combined Batches

Preparation method, cell line	Sensitivity	Specificity	Accuracy
Cryosection, 1	0.85	0.70	0.82
Cryosection, 2	0.91	0.99	0.98
Cryosection, combined	0.78	0.55	0.66
FFPE, 1	0.99	0.99	0.99
FFPE, 2	0.83	0.85	0.92
FFPE combined	0.95	0.89	0.97

FFPE = formalin-fixed paraffin-embedded.

## Discussion

We have demonstrated that Raman spectroscopy can determine the most common genetic subtypes of adult diffuse gliomas using fresh tissue straight from the operating theatre, cryosections, and FFPE sections. Most significantly, our fresh tissue workflow requires no sample processing, and results are available in under 15 min, making this technique ideal for rapid intraoperative diagnosis.

Surgery plays an important role in the treatment of gliomas. There is mounting evidence that the greater the extent of surgical resection the longer the progression-free and overall survival.^1–3^ The aim of surgery is to remove all the contrast-enhancing tumor in high-grade tumors or all the T2-FLAIR signal in low-grade gliomas, but, due to their diffuse nature, it is known that tumor cells will have infiltrated beyond these boundaries. Removal of the brain surrounding the tumor may result in neurological disability for the patient. The crucial balance for the surgeon is to maximize resection of the tumor whereas minimizing the risk of disability. Surgeons vary widely as to how aggressively they pursue tumor resection and the techniques they use (such as awake surgery and white matter tractography) to define this tumor:functional boundary. This is reflected in a wide range of 15%–70% complete resection rates reported in the literature.^2,17^ In this era of personalized medicine, the ability to perform accurate genetic subtyping of gliomas at the time of surgery will allow the surgeon to adapt their surgical strategy with some knowledge of the likely prognostic and treatment potential for the lesions. In this study, we have shown that Raman spectroscopy can accurately identify the 3 most common and clinically relevant glioma groups. The presence of an IDH mutation is a strong predictor of longer progression-free survival^18–20^ and overall survival^18–25^ when compared with IDH-wild-type tumors. 1p/19q-codeletion in the context of an IDH mutation is the genetic hallmark of oligodendrogliomas^26^ and is a strong favorable prognostic factor for overall survival^20,23,27^ and a strong predictor of response to radiochemotherapy.^28–31^ More relevant to intraoperative decision-making, Wijnenga et al.^2^ examined the impact on overall survival of the extent of surgical resection, measured as volume of residual tumor seen on postoperative contrast magnetic resonance imaging, in molecularly stratified low grade gliomas (WHO grade II astrocytomas and oligodendrogliomas). They reported that the effect of less tumor residual volume on overall survival was much greater in IDH-mutant astrocytomas compared with oligodendrogliomas. In the IDH-mutant astrocytoma group, even a small amount of residual tumor of 0.1 cm^3^ affected negatively on survival compared with 0 cm^3^ residual. This was not seen in the oligodendroglioma group, with a small residual having no significant effect on overall survival. Similar finding was reported by Kawaguchi et al.^32^ who found a significant survival advantage of gross total resection over subtotal resection in IDH-mutant astrocytoma but no significant survival advantage for oligodendroglioma or IDH-wild-type astrocytoma. Consequently, pushing the boundaries of surgical resection when there is a possible increased risk of disability might be in the patient’s best interest if they have an IDH-mutant astrocytoma but would not be in their interest if they have an oligodendroglioma: intraoperative information of the glioma genetic subtyping is therefore essential. As we learn more about glioma subtypes and develop novel therapies tailored for their treatment, including the insertion of therapy agents at the time of surgery, the need for accurate genetic subtype information at the time of surgery will increase. We, and others, have shown that preoperative non-invasive magnetic resonance spectroscopy at 3- or 7-Tesla may provide the neurosurgeon with information on IDH status of diffuse gliomas^33^; however, to the best of our knowledge, this currently does not allow distinction between IDH-mutant astrocytomas and oligodendrogliomas.

One aim of our study was to build up the training dataset for model development and therefore true intraoperative classification was not possible until enough data had been acquired. Once a classification model was developed, samples could be analyzed and classified immediately intraoperatively. This was piloted in the last 5 cases collected and yielding similar classification performances to the results presented. The major advantage of Raman spectroscopy compared with conventional techniques is the lack of sample preparation required. We developed a protocol that could easily be replicated by anyone in the operating theatre. The fresh tissue samples simply require the biopsy to be smeared into a grooved slide and then placed on the Raman microscope stage (Figure 2). In our study protocol, the sample was split into 2 sections by the neuropathologist before Raman analysis, as adjacent intraoperative or permanent tissue staining was essential to establish the quality of the biopsy and tumor content, and spectral collection was supervised by a researcher. These steps would not be required as part of a Raman diagnostic pathway, particularly with the straightforward automation of the process, which, at the press of a button, would perform spectrometer quality control, laser autofocusing, spectral acquisition, preprocessing, and classification. This sets Raman spectroscopy apart from other techniques that have been used to acquire intraoperative genetic subtyping, such as polymerase chain reaction-based rapid genotyping assays.^34,35^ More traditional techniques can give genetic stratification within the timeframe of an operation and are more accurate than Raman spectroscopy, but they all require complex laboratory-based sample processing and analysis by qualified laboratory technicians. They have significant consumables costs per test, compared with the minimal cost of the grooved stainless steel slide used for Raman analysis, which after the procedure could be sterilized, reused, and become part of the standard neurosurgical instrument set. Others have reported the use of Raman and attenuated total reflectance-Fourier transform infrared spectroscopy spectroscopy to distinguish IDH-mutant versus IDH-wild-type but have used snap frozen sections before spectroscopy, which still requires laboratory-based sample preparation.^36,37^ These vibrational spectroscopy studies have not attempted to distinguish oligodendrogliomas as a separate group, which is essential for clinical decision-making. Raman spectroscopy has also been used intraoperatively in the form of a probe device to attempt to differentiate tumor boundaries.^38^ A probe device is essential for boundary detection but adds no benefit to the aim of this study: as the tumor is being removed by the surgeon anyway, in- or near-operating theatre analysis is sufficient and allows for more sensitive Raman spectrometry than would be achievable via a fiber optic probe setup.

A barrier to the translation of Raman spectroscopy into the clinical setting is that clinicians are skeptical about the reliance on a change in spectral patterns to define different pathological entities. The spectral characteristics of glioma found in this study add to a body of published evidence, which consistently defines similar key spectral features of glioma tissue.^15,16,36,39,40^ Moreover, the most significant spectral differences seen between genetic subgroups are broadly consistent with our understanding of the biology of these tumors. IDH-mutant and IDH-wild-type gliomas are known to have significant differences in lipid metabolism,^41–44^ collagen maturation,^45^ and DNA methylation,^7,46^ which correlates with the most differentiating Raman peaks assigned to lipids, phenylalanine (involved in collagen synthesis), and DNA. The 881 cm^–1^ peak is markedly different in the fresh tissue oligodendroglioma spectrum. We currently do not have an experimentally validated explanation for this; however, although superficially similar (regarding high levels of 2HG as a result of an IDH mutation), IDH-mutant astrocytomas and oligodendrogliomas are otherwise clinically and molecularly distinct. It is also known that certain metabolic features are associated with the 1p/19q codeletion rather than IDH mutation (eg, cystathionine^47^). As altered phospholipid metabolism is known to occur in subsets of glioma,^48^ we cannot rule out that the increased peak at 881 cm^–1^ reflects these metabolic changes rather than an increased amount of DNA. It is important to note that this study did not aim to investigate proteomic or metabolomic profiles of different genetic subtypes—there are more suitable techniques to do this than Raman spectroscopy.

There is considerable consistency of Raman spectral peaks across all of the different sample preparation methods and cell lines used in this study. As might be expected, the performance of the classification model decreases as the tissue becomes more manipulated by the sample preparation, with fresh tissue models performing better than cryosection, which in turn classifies better than FFPE sections. Spectra of cryosections and FFPE sections have fewer contributions from proteins and lipid, differentiating genetic subtypes more on DNA and amino acid peaks. This is consistent with the known effect of protein denaturing due to freezing, cross-linking of proteins as part of formalin fixation, and the loss of lipid due to paraffin embedding.^49^

The LN18 cell line classification was very accurate within individual cell line preparations. In the cryosection preparations, the performance dropped off when the model includes spectra from both cell lines. This may be the result of large intragroup variability, as there are only 2 groups of each mutation and wild-type cells in the model. The performance would be likely to increase when the number of cell line batches is much greater, causing a decrease in intragroup variability and an increase the intergroup variability.

This study has also shown that Raman spectroscopy has application in the neuropathology and research laboratory settings, offering rapid genetic subtyping of gliomas using cryosections, FFPE sections, and glioma cell lines. The minimal sample preparation and rapid analysis advantages of Raman spectroscopy are less critical within a laboratory setting. However, routine IHC testing for IDH mutation is only sensitive to the common IDH1 mutation,^7^ whereas Raman spectroscopy measures the downstream biological consequences of the mutation, most likely associated with the intratumor accumulation of 2-hydroxyglutarate, which is common to all glioma-associated IDH1 and IDH2 mutations.^50^ In our study, the IHC test for the common IDH1-R132H mutation was, as expected, negative in those cases harboring rare IDH1 or IDH2 mutations (Table 3). This amounted to a significant proportion of our cohort (5%) and all cases were correctly classified as IDH-mutant in the Raman analysis. High-throughput assays based on next-generation sequencing or microarray methylation chip techniques are likely to replace single marker assessment by IHC, Sanger sequencing, or FISH, but these are not currently widely available and have a 5- to 14-day turnaround. Raman analysis may provide a faster and lower-cost alternative, certainly for initial tissue screening in environments where access to molecular diagnostics may not be available.

In summary, this study has demonstrated the potential for Raman spectroscopy to be used in a straightforward way within the neurosurgical and neuropathological glioma diagnostic pathway to provide genetic subtyping of gliomas within 15 min of the biopsy. The resulting data give the surgeon the opportunity to tailor the surgical strategy to the patient’s specific tumor genetics. Despite this being the largest study of its kind published to date, the small sample size is a limitation. Further multicenter and larger studies need to be undertaken to expand the training data set and to evaluate the effect of inter-user and inter-machine variability on the predictive model. Finally, the predictive model needs to be tested with a prospective independent cohort of patients to robustly validate the technique.

## Supplementary Material

vdz008_suppl_Supplementary_Materials
