# Supplementary material for: Rapid intraoperative molecular genetic classification of gliomas using Raman spectroscopy
Source: Neurooncol Adv. 2019 May 28;1(1):vdz008. doi: 10.1093/noajnl/vdz008 (PMC6777649; doi:10.1093/noajnl/vdz008)
Supplement: vdz008_suppl_Supplementary_Materials [file vdz008_suppl_supplementary_materials.docx]

**Supplementary material**

**METHOD**

**Pathological diagnosis**

IHC staining was carried out for the common IDH mutation (IDH1-R132H) and nuclear ATRX. FISH analysis for 1p/19q-codeletion was undertaken in cases that were IDH mutant and retained ATRX. As per EANO guidelines, IDH-wildtype and ATRX-retained cases in patients over 55 years old with characteristic glioblastoma histology were diagnosed as glioblastoma, IDH-wildtype, as the possibility of a rare IDH mutation being present is extremely low. In our laboratory we also routinely undertake IHC for BCAT1 (branched-chain amino acid transaminase 1). BCAT1 up-regulation has been strongly associated with IDH wildtype tumors^10^, in cases negative for IDH1-R132H IHC with diffuse over-expression of BCAT1 in tumor cells, the likelihood of a rare IDH mutation is close to zero (OA, unpublished observations n=200). In all cases where BCAT1 is not up-regulated, or where there is histological or clinical ambiguity, the samples underwent targeted genetic sequencing for IDH1, IDH2, Histone H3F3A and BRAF V600E. Tumors that did not fit into one of the above categories (e.g. “Diffuse midline glioma” or “glioma, not otherwise specified (NOS)”) were excluded from the study.

**Tissue processing - Cryosection preparation protocol**

Two parallel 10 µm sections were cut from selected samples using a cryotome. One section was mounted on a glass slide and underwent standard H&E staining. The second section was mounted on an APTES ((3-Aminopropyl)triethoxysilane, Sigma-Aldrich, UK) coated stainless steel slide and returned to the -80 °C freezer. When Raman measurements were to be undertaken, these sections were removed from the freezer and allowed to thaw for 15 minutes. H&E sections were reviewed by OA, and areas of tumor identified. These areas were then identified on the unstained parallel section on the stainless steel slide, and Raman data were recorded in these locations. If the H&E sample did not demonstrate evidence of tumor (usually indicating normal brain, necrosis or haemorrhage) then the Raman sample was excluded from the study.

**Tissue processing - FFPE section preparation protocol**

Two parallel 6 µm sections were cut from selected cases using a microtome. One section was mounted on a glass slide and underwent standard H&E staining. The second section was mounted on an APTES coated stainless steel slide. These were then placed in an oven at 40 °C for 30 minutes to increase sample adherence to the slide. Following this, samples were deparaffinised using Histo-Clear (National Diagnostics, USA) incubations for 3 x 5 minutes. Samples were then incubated with 100% isopropyl alcohol (IPA) for 2 x 10 dips, rehydrated (90% IPA for 10 dips, 70% IPA for 10 dips, distilled water for 10 dips) and allowed to air dry before being stored in a slide box until they were used for Raman analysis. Similar to the cryosections, H&E sections were reviewed by OA and areas of tumor identified and located on the unstained stainless steel section. Samples not demonstrating evidence of tumor were excluded from the study.

**Tissue processing - LN18 cell line preparatation protocol**

Mutant R132H human IDH1 was cloned into a transfer plasmid of lentiviral vectors which were then transduced into the LN18 parental cells (ATCC, UK). The LN18 transduced cells over-express IDH-R132H transgene compared to the IDH wildtype parental cells. Mutated and parental cell lines were then grown in parallel for two weeks in Dulbecco’s modified Eagle media prior to harvesting. Cells were detached using trypsin to keep cells intact and then washed with PBS. The cells were divided into two batches: one for cryosections and one for FFPE sections, with the aim of establishing protocols similar to those used for tissue sample preparation. Cells destined for FFPE sections were placed directly in 10% formalin for 12 hours after harvesting. They were then centrifuged (2000 rpm for 5 minutes) to form a pellet, which was transferred to an embedding capsule and embedded in paraffin overnight. Three parallel sections of 6 μm thickness were cut from the cell paraffin blocks. One section was placed on a stainless steel slide for Raman analysis and underwent the same deparaffination and preparation as the FFPE tissue sections. Of the remaining two sections, one underwent H&E staining and one IDH1-R132H immunohistochemistry to confirm the presence or absence of the mutation in the cell cultures. Cells destined for cryosection preparation were suspended in phosphate buffer solution after harvest and centrifuged (1300 rpm for 5 minutes) to form a pellet. The supernatant was removed and an embedding medium (OCT (CellPath Ltd, UK)) added to the Eppendorf tube and allowed to settle at the bottom over the cell pellet. The pellet was snap frozen using isoprolene cooled with liquid nitrogen for 1 to 2 minutes. Once frozen, the Eppendorf tube was cut with a hacksaw just above the frozen pellet, which was then removed and mounted on cork with the embedding medium. The mounted pellet was snap frozen again in isoprolene and then stored in a -80 °C freezer. Three parallel sections of thickness 10 μm were cut from the cell cryoblocks. One section was placed on a stainless steel slide for Raman analysis as per the cryosection tissue samples. The other two sections were mounted on standard glass slides and underwent H&E staining or IDH1-R132H immunohistochemistry.

**Raman spectroscopy**

Raman spectra were collected using a Renishaw bench-top RA800 series spectrometer (Renishaw plc, UK) equipped with an ×50 objective (Nikon TU Plan Fluor EPI 50X objective, 0.8 NA) and a 785 nm excitation laser with 180 mW power at the objective. The spectrometer has automated auto-alignment and self-calibration sequences, based on built-in neon and silicon references. These were employed for all Raman data collected for this work. Data acquisition parameters were controlled using WiRE v4.3 software (Renishaw, UK). Acquisition parameters were optimised for each tissue type to achieve the highest possible signal to noise ratio without detector saturation, tissue damage, or change in spectral characteristics due to laser exposure. Preliminary experiments using all tissue types (fresh, cryosection, FFPE) and LN18 cells were undertaken to establish the optimum laser power, acquisition time, number of accumulations and map area geometry and step size.

For cryosections and FFPE samples, two or three (depending on the size of sample available) 500 µm^2^ areas were chosen corresponding to the area identified by the Neuropathologist on the parallel H&E section as containing tumor. A rectangular grid of 66 single spectra were acquired over each chosen area. The acquisition comprised two accumulations of 5 seconds each for cryosections and two accumulations of 1 second for FFPE sections. For the LN18 cells, five 500 µm^2^ areas of high cell density were chosen, with acquisition times the same as for the respective tissue cryosections and FFPE sections.

**Statistical analysis and model building**

Prior to analysis, the data were filtered to exclude spectra which did not meet a specified signal-to-noise threshold (low signal or high fluorescence) and cosmic ray artefacts were removed using a standard deviation threshold. In no case were more than 30% of the spectra removed by this filtering. Spectral baseline correction and normalisation, to account for variations in acquisition parameters and tissue fluorescence, was carried out using Extended Multiplicative Scatter Correction (EMSC). EMSC is a model-based preprocessing method regularly used in vibrational spectroscopy1. The EMSC model used in this study assumed each spectrum to be made up of 1) a background signal; 2) the spectral contribution of the objective lens; 3) the mean spectrum of all the dataset and 4) the unique aspects of the spectrum of interest. The background signal was removed using polynomial background correction (a third-order polynomial was used throughout this study) along with removal of the objective lens contribution. The overall mean spectrum was then subtracted to normalise the spectrum, yielding a signal that reveals the unique aspects of the spectrum of interest. All preprocessing steps were evaluated independently to assess their effect on the model performance in an attempt to minimise the risk of overfitting of the model. The Raman spectral region between 400 and 1850 cm^-1^ was analysed, excluding that from 1560 to 1578 cm^-1^, which is affected by the Raman band of oxygen. The mean spectrum of each genetic subtype was obtained and normalised using EMSC to allow comparison. The second-derivative transformation of the mean spectrum was derived in order to better resolve the Raman peaks^32^, and the negative of the second derivative was plotted against the wavenumber shift. For each wavenumber at which a Raman peak was identified from the mean spectra, the normalised intensities of all spectra of a given genetic subtype were analysed and compared with the intensities of the other genetic subtype(s) in the model. In both the two-group and three-group models, IDH-mutant intensities were compared with IDH-wildtype intensities. Additionally, in the three-group model, oligodendroglioma intensities were separately compared with astrocytomas, IDH-mutant and astrocytomas, IDH-wildtype intensities.

Leave-one-patient-out cross-validation was used in this study. All the data from one patient is omitted, a model is built with the data from the remaining patients (n=61 in the case of the fresh tissue model) and then each spectrum from the omitted patient is classified by the model. This process is then repeated omitting a different patient each time giving a total of n classification models. The sensitivity, specificity and area under ROC curves are then calculated using the combined performance of each independent models. This is independent validation because no spectra from the same patient is used in the same model for both model building and classification.

**RESULTS**

In the cryosection group, 15,576 spectra were collected from 236 different 0.5 mm^2^ regions on the sections (66 spectra per region). Each region took approximately 13 minutes to complete. In the FFPE sections, 23,760 spectra were collected from 360 0.5 mm^2^ regions (66 spectra per region). Each region took approximately 3 minutes 30 seconds to collect.

**RESULTS** - **Supplementary Figures**

**Sup figure S1.** A) Cryosections and B) FFPE sections 3-group model. i) Mean spectra and ii) negative of second derivative transformation of mean spectra for each genetic subtype. The wavenumber of prominent Raman peaks are labelled in B and the statistical significance of the difference between the peak intensities of each genetic subtype in that model is indicated (p <0.01) for *IDH-mutant versus IDH-wildtype; ͂͂ astrocytoma, IDH-mutant versus oligodendroglioma; and ⁺ astrocytoma, IDH-wildtype versus oligodendroglioma. (Astro MUT = astroglial tumour IDH mutant; Astro WT = astroglial tumour IDH-wildtype; Oligo = Oligodendroglioma).


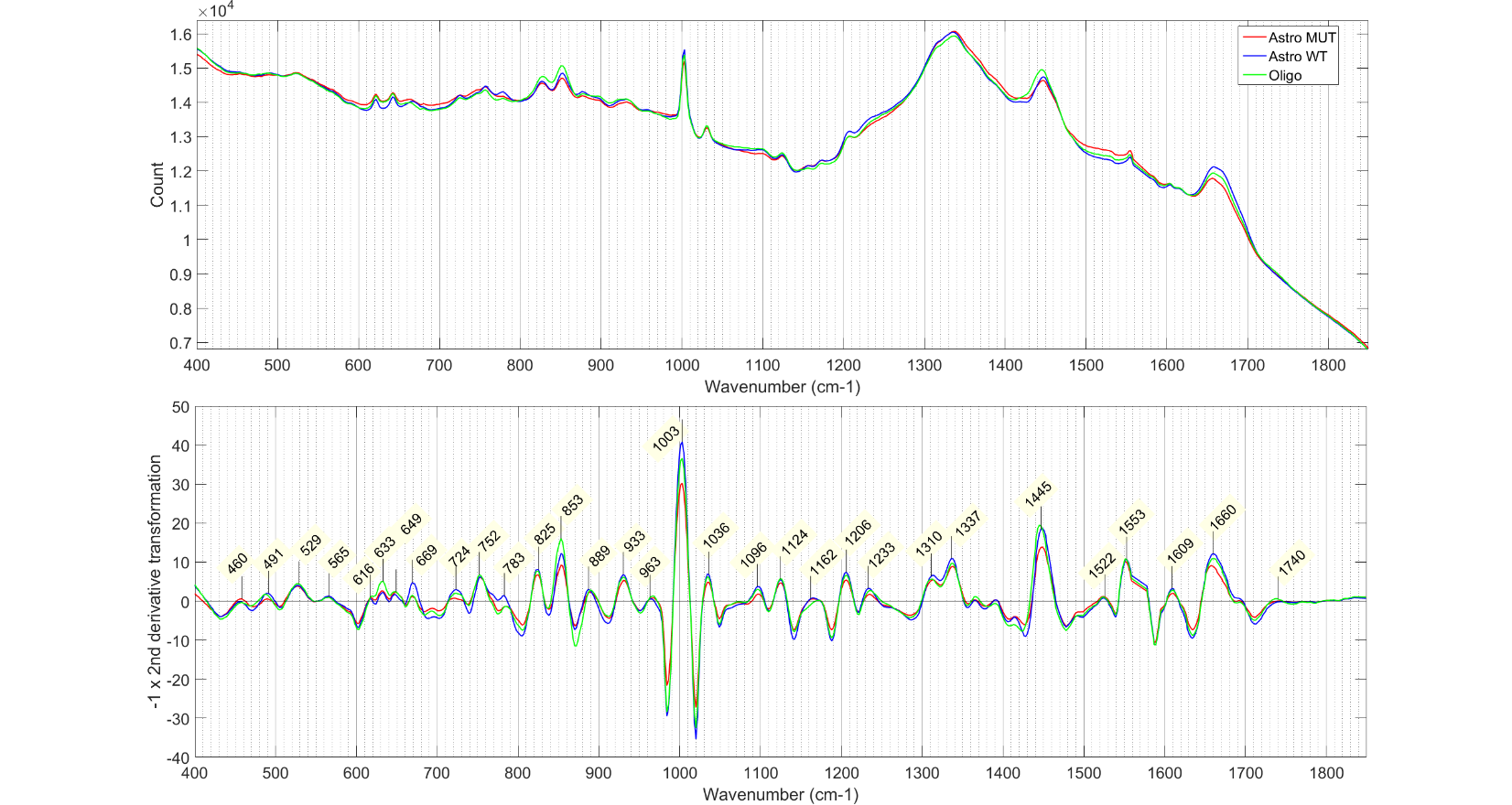


* ͂͂

* ͂͂⁺

* ͂͂

* ͂͂⁺

* ͂͂⁺

* ͂͂⁺

* ͂͂⁺

* ͂͂⁺

͂͂⁺

* ͂͂⁺

* ͂͂⁺

* ͂͂⁺

* ͂͂⁺

* ͂͂⁺

* ͂͂

* ͂͂⁺

* ͂͂⁺

*

* ͂͂⁺

* ͂͂⁺

* ͂͂⁺

* ͂͂⁺

* ͂͂⁺

* ͂͂⁺

* ͂͂⁺

* ͂͂⁺

* ͂͂⁺

* ͂͂⁺

* ͂͂⁺

* ͂͂⁺

* ͂͂⁺

i

ii

A


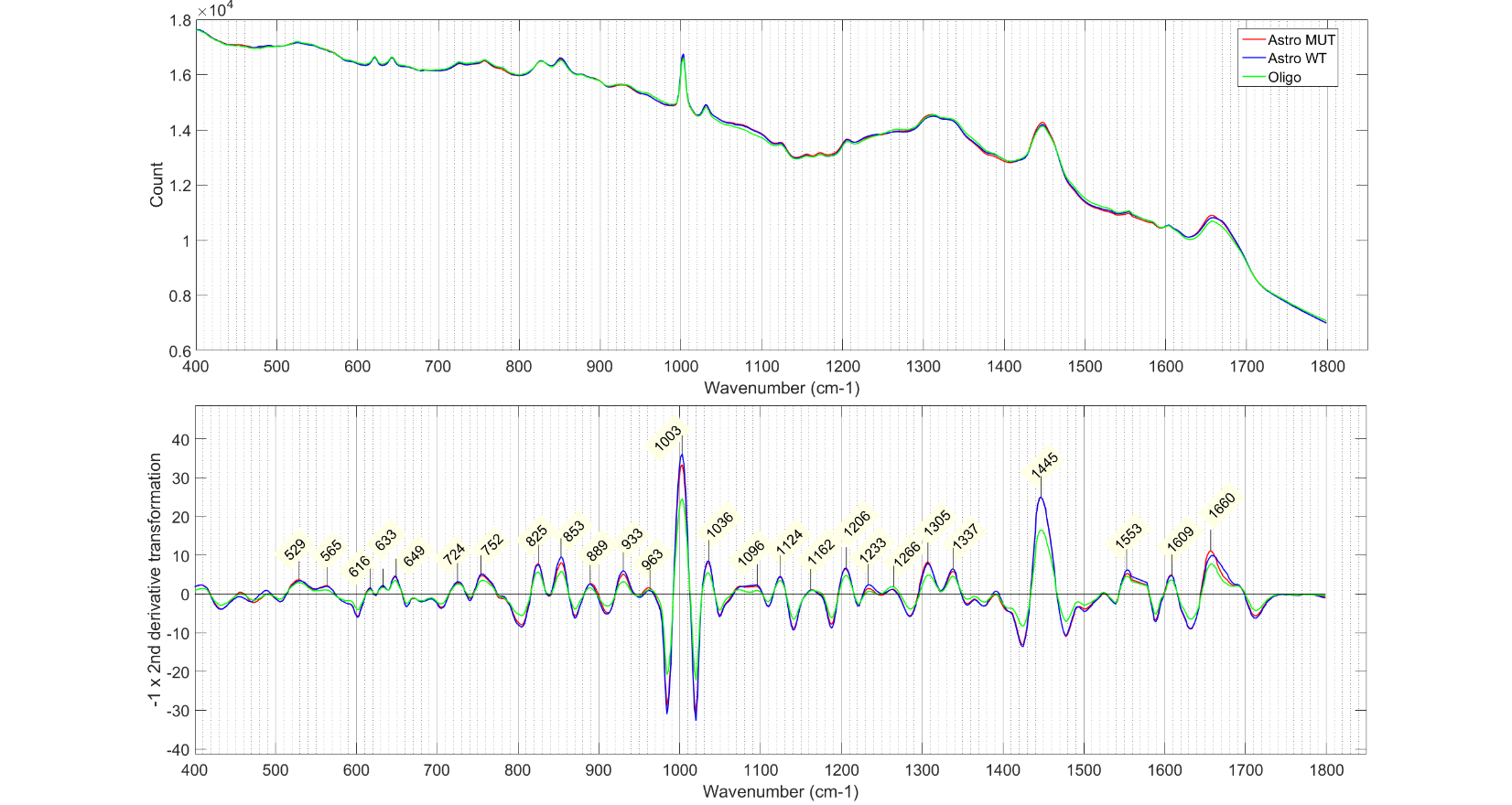


* ͂͂⁺

͂͂⁺

* ͂͂⁺

* ͂͂⁺

͂͂⁺

* ͂͂⁺

* ͂͂⁺

* ͂͂⁺

* ͂͂⁺

* ͂͂

* ͂͂⁺

* ͂͂⁺

* ͂͂⁺

* ͂͂⁺

͂͂⁺

* ͂͂

* ͂͂⁺

* ͂͂⁺

* ͂͂⁺

* ͂͂⁺

* ͂͂⁺

* ͂͂⁺

* ͂͂⁺

*⁺

* ͂͂⁺

* ͂͂⁺

i

ii

B

**Sup figure S2.** A) Cryosection and B) FFPE 2-group model. i) Mean spectra and ii) negative of second derivative transformation of mean spectra for each genetic subtype. The wavenumber of prominent Raman peaks are labelled in B and the statistical significance of the difference between the peak intensities of each genetic subtype in that model is indicated (p <0.01) for *IDH-mutant versus IDH-wildtype. (IDH-MUT = IDH-mutant; IDH-WT = IDH-wildtype).


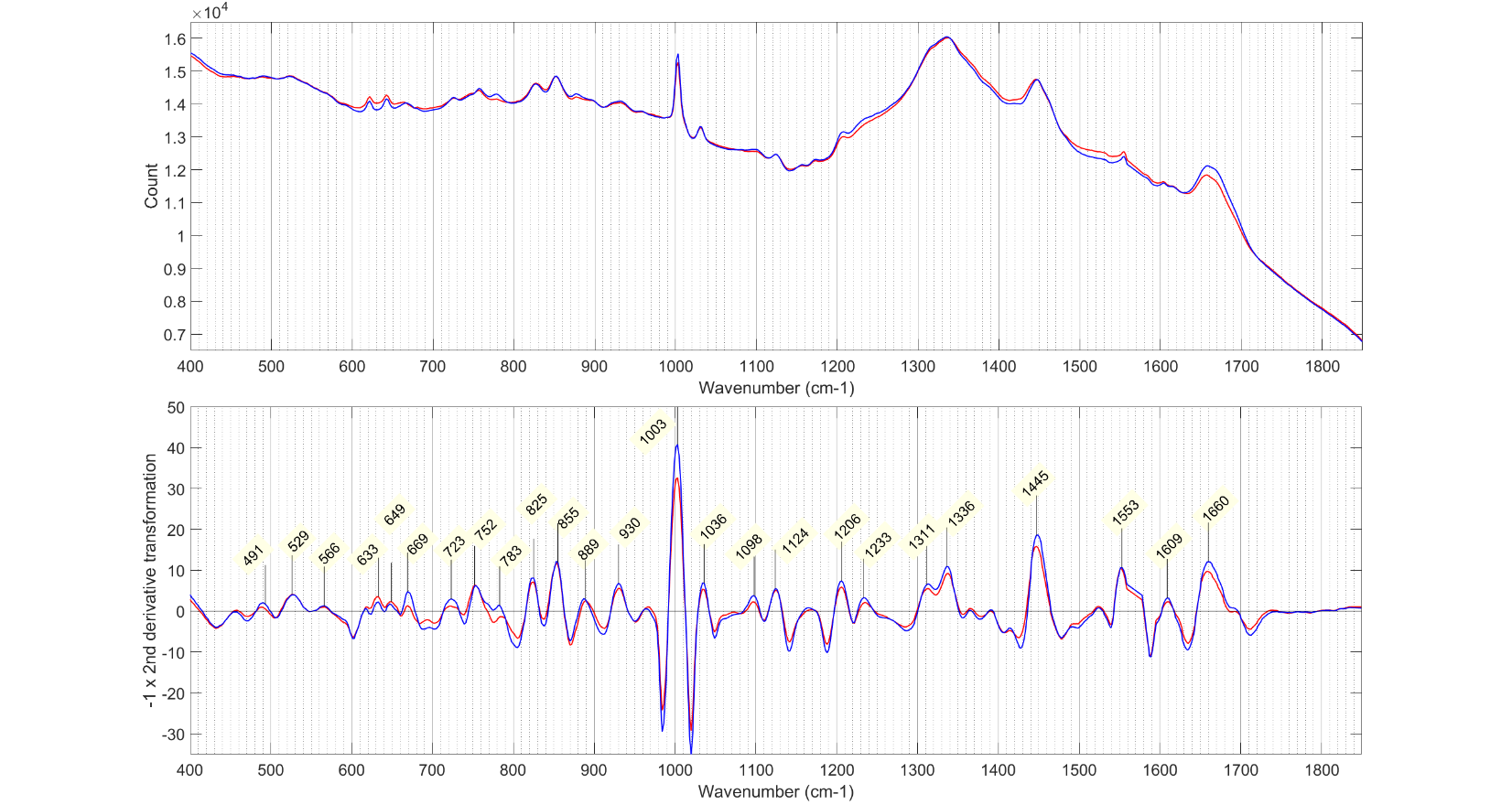

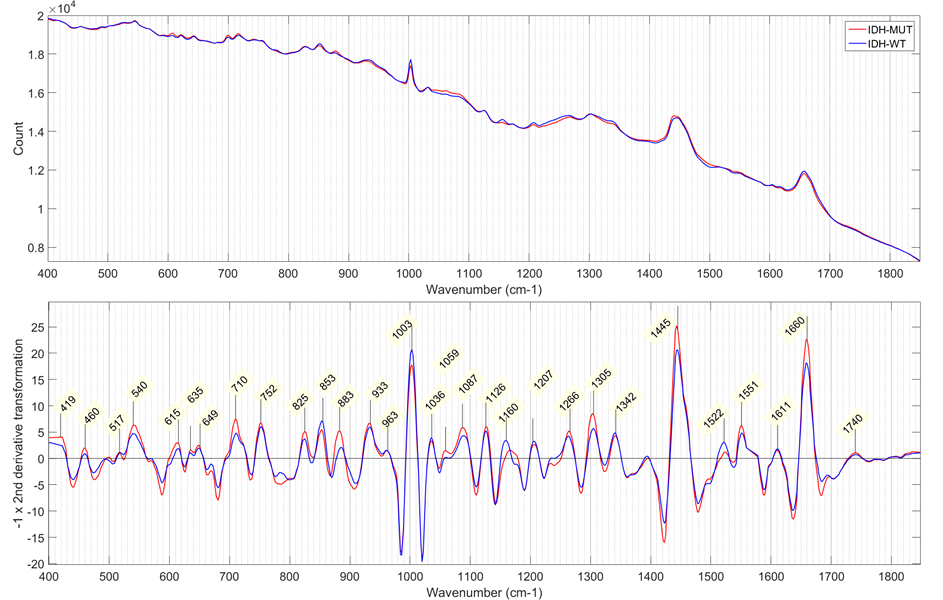


*

*

*

*

*

*

*

*

*

*

*

*

*

*

*

*

*

*

*

*

*

*

i

ii

A


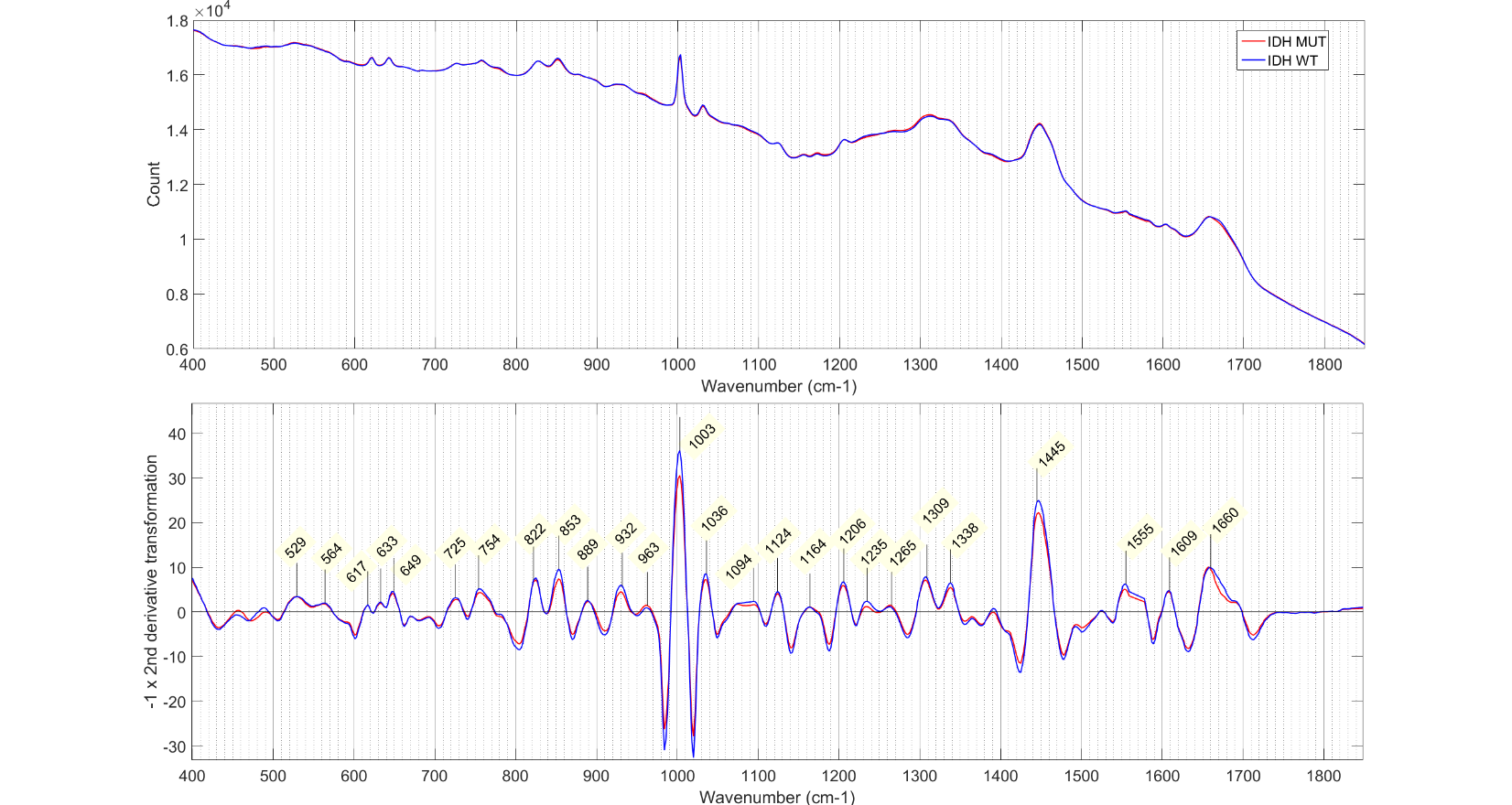


*

*

*

*

*

*

*

*

*

*

*

*

*

*

*

*

*

*

*

*

*

i

ii

B

**Sup figure S3.** LN18 cell line A) Cryosection and B) FFPE 2-group model. i) Mean spectra and ii) negative of second derivative transformation of mean spectra for each genetic subtype. The wavenumber of prominent Raman peaks are labelled in B and the statistical significance of the difference between the peak intensities of each genetic subtype in that model is indicated (p <0.01) for *IDH-mutant versus IDH-wildtype. (IDH-MUT = IDH-mutant; IDH-WT = IDH-wildtype).


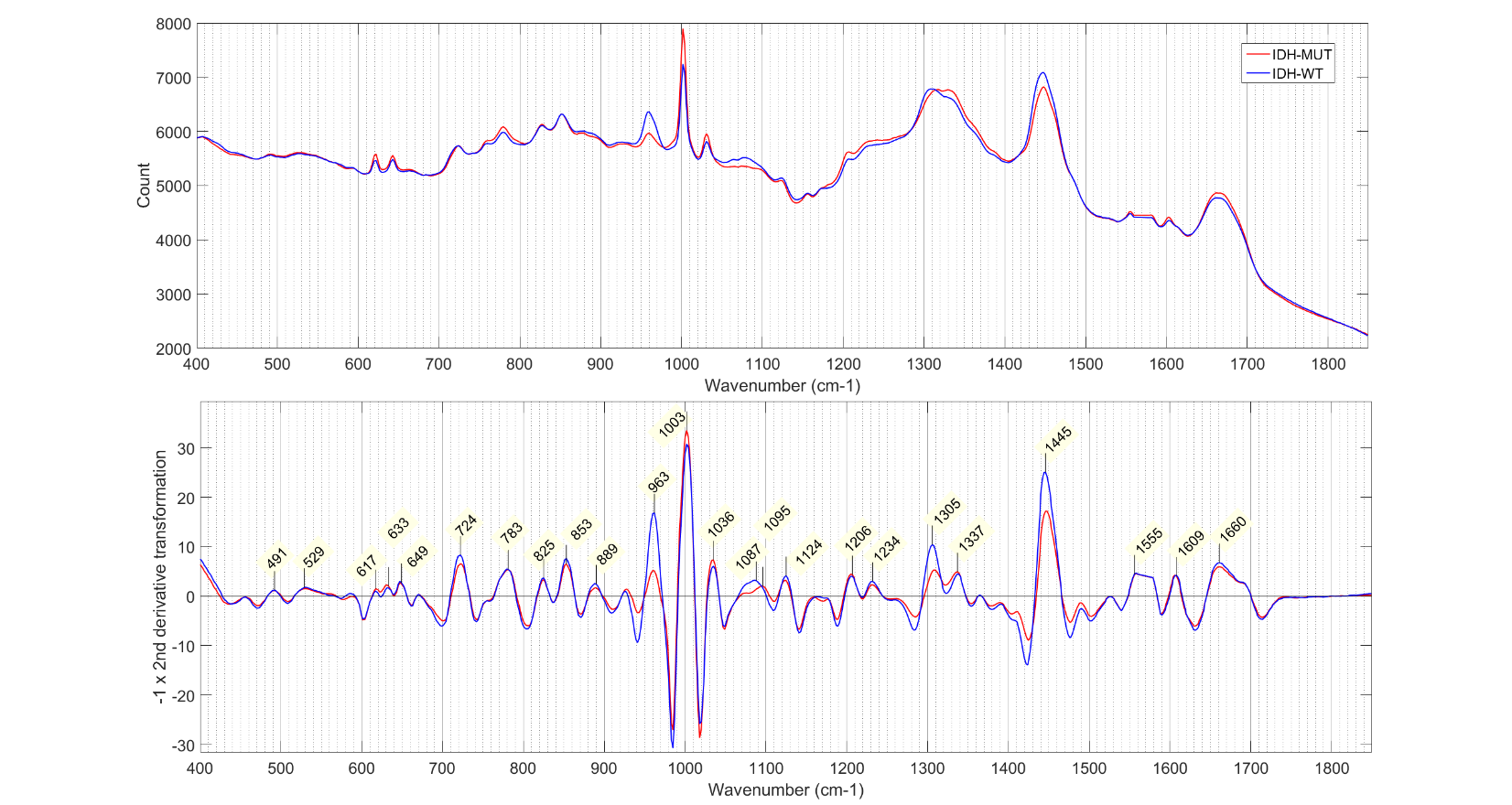


*

*

*

i

ii

B

*

*

*

*

*

*

*

*

*

*

*

*

*

*

*

*

*

*

*


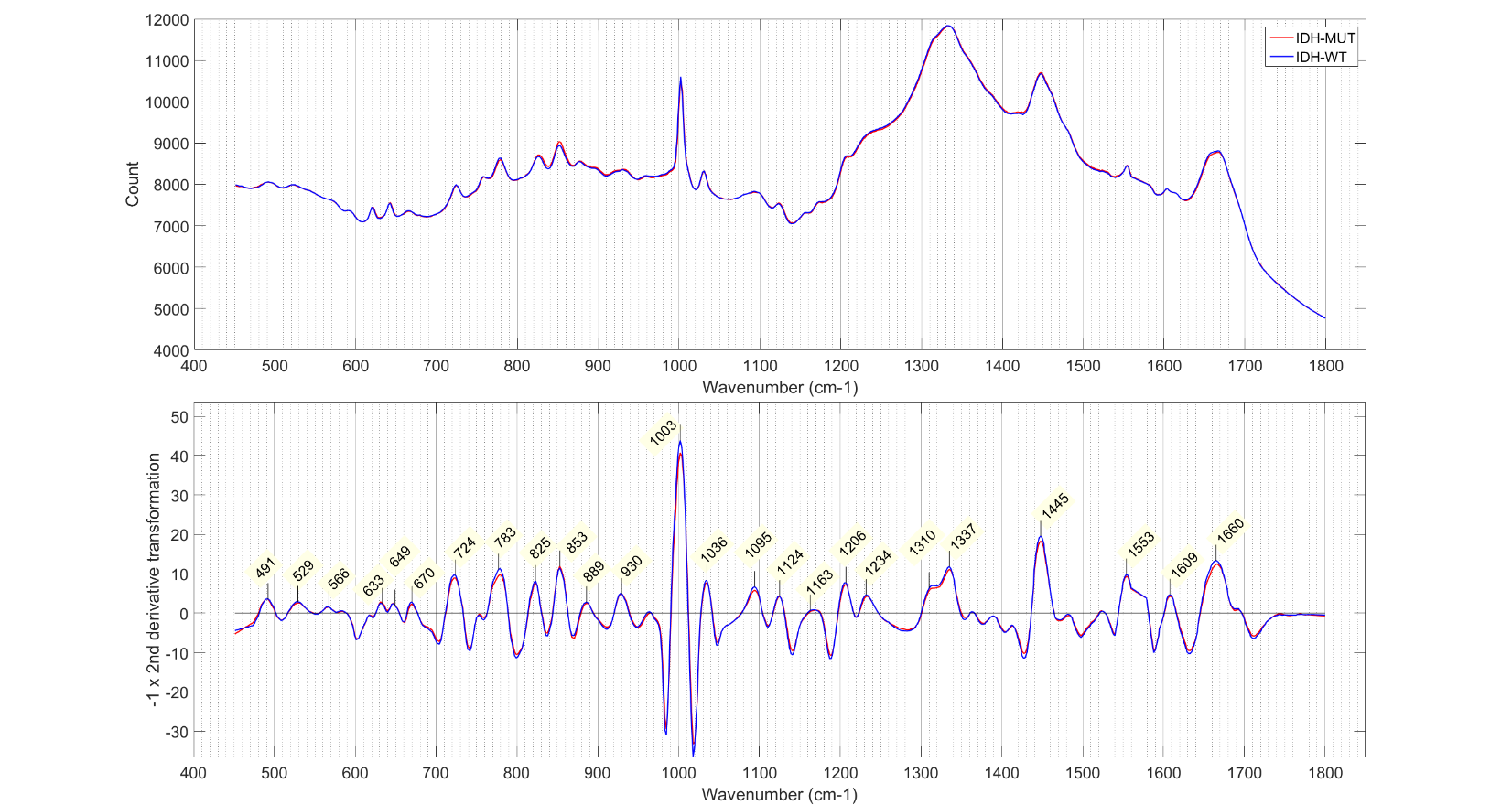


*

*

*

i

ii

A

*

*

*

*

*

*

*

*

*

*

*

*


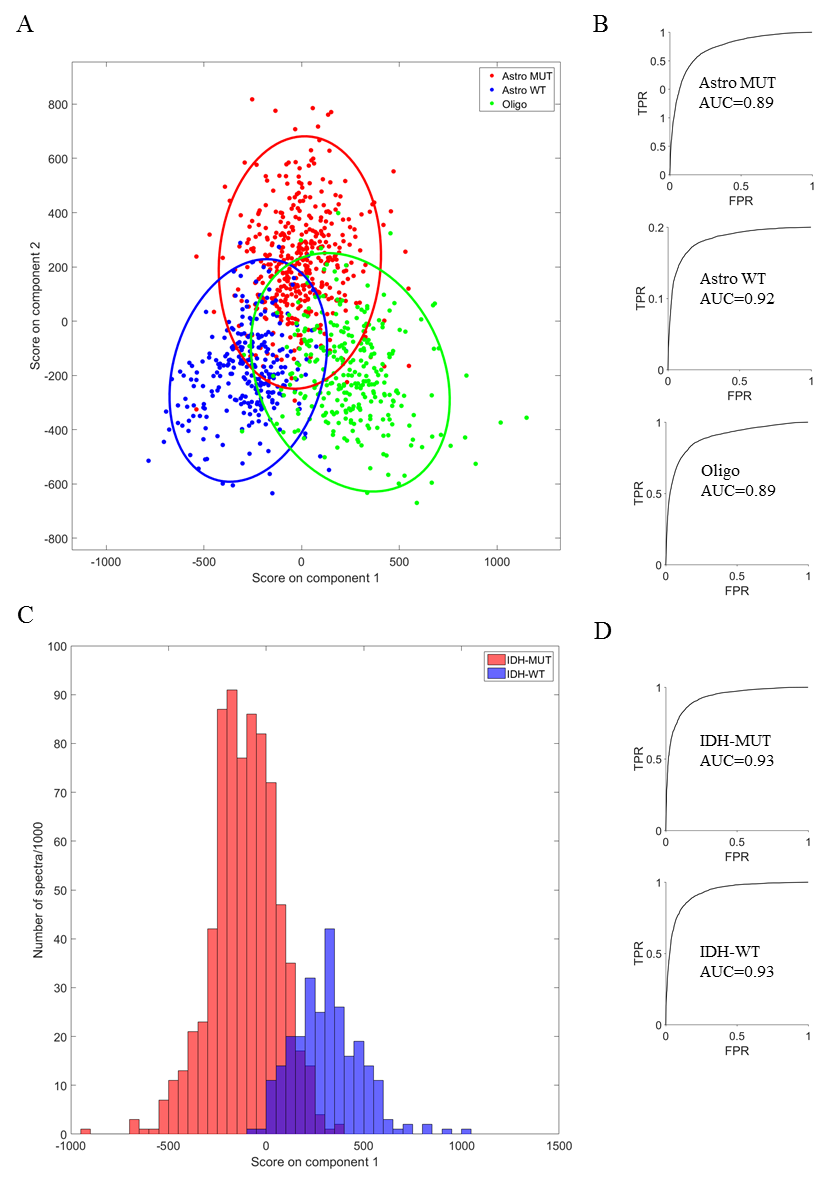
**Sup figure S4.** Cryosection LDA score plots and ROC curves for 3-group model (A, B) and 2-group model (C, D). (TPR= true positive rate; FPR= false positive rate; Astro MUT = astroglial tumour IDH-mutant; Astro WT = astroglial tumour IDH-wildtype; Oligo = Oligodendroglioma).


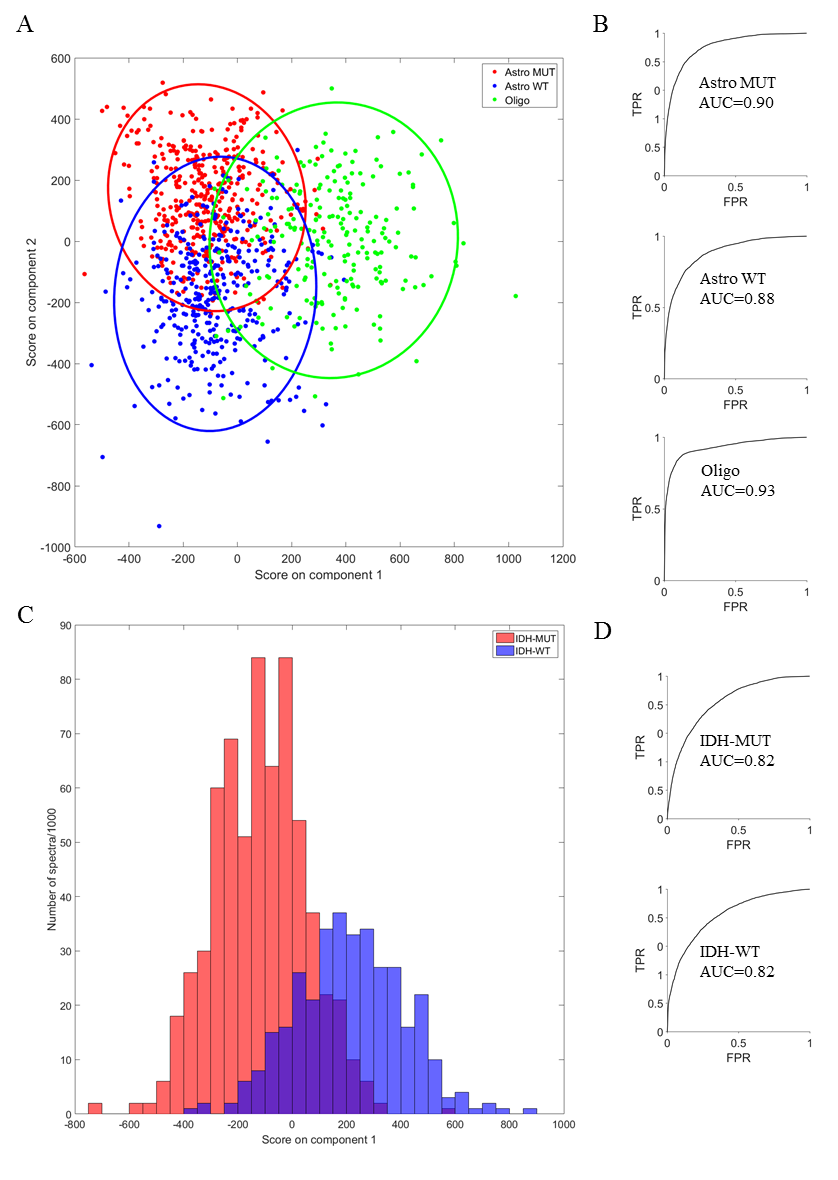
**Sup figure S4.** FFPE section LDA score plots and ROC curves for 3-group model (A, B) and 2-group model (C, D). (TPR= true positive rate; FPR= false positive rate; Astro MUT = astroglial tumour IDH mutantd; Astro WT = astroglial tumour IDH-wildtype; Oligo = Oligodendroglioma).
